# Necrotizing Fasciitis in COVID-19 Patient: A Case Report of Atypical Early Presentation

**DOI:** 10.7759/cureus.37290

**Published:** 2023-04-08

**Authors:** Amro Alhoukail, Talal Alrawaf, Abdullah Alotaibi, Hassan Alanazi

**Affiliations:** 1 Department of Orthopedic Surgery, King Fahad Medical City, Riyadh, SAU

**Keywords:** atypical infection, nf, surgery, streptococcus, necrotizing fasciitis

## Abstract

A 48-year-old male, known to have hypertension (HTN), ischemic heart disease (IHD) post-percutaneous coronary intervention (PCI) before one year, and morbid obesity (BMI: 60), presented to the emergency department complaining of right thigh pain and swelling that started two days before. The swelling got increasingly worsen over the previous days, associated with dyspnea, for which he sought medical attention in another hospital. He was found to have a picture of sepsis where they offered him irrigation and debridement (I&D) but he refused and presented to our institution in a hemodynamically unstable condition. The patient underwent immediate surgery with subsequent intensive care unit (ICU) admission as a case of necrotizing fasciitis complicated by sepsis. Later he was found to have coronavirus disease 2019 (COVID-19) infection.

## Introduction

Necrotizing fasciitis (NF) is a rare but potentially life-threatening and fatal infection involving the subcutaneous tissue and fascia. It is commonly known as flesh-eating disease. Deaths from NF can be surprisingly quick and sudden due to the rapid progression of infection. Necrotizing fasciitis is prevalent enough that most primary care physicians will be involved with managing at least one case during their time in practice, but infrequent enough for most to be unfamiliar with the disease [1].

NF is a very serious bacterial infection. The bacteria multiply and release toxins and enzymes that result in soft tissue necrosis and thrombosis in the blood vessels. The result is the destruction of the soft tissues and fascia [2]. It is an uncommon disease that results in gross morbidity and mortality if not recognized and treated in its early stages [1].

There are many types of bacteria that can cause the “flesh-eating disease” so-called necrotizing fasciitis. Public health experts believe group A Streptococcus (group A strep) is the most common cause of monomicrobial necrotizing fasciitis [3]. Although predisposing etiologies are many, they mostly center on impaired immunity occurring directly or indirectly and loss of integrity of protective barriers which predispose to infection. The non-specific or atypical patient presentation in NF may delay the diagnosis and lead to high morbidity and mortality [4,5].

It is a rare disease with an annual incidence of about 1.5 cases/100,000 population; but the mortality is rather high, reaching about 20-30% [6]. Clinical presentations are usually seen with skin discoloration, palpable crepitus, and bullae with systemic manifestations of sepsis on delayed basis. Risk factors include compromised integrity of skin or mucous membranes, diabetes, arteriopathy, alcoholism, obesity, immunosuppression, malnutrition, renal failure, and age > 60 years. Non-steroidal anti-inflammatory drugs have been suggested as possible risk factors for NF [1,7].

The Laboratory Risk Indicator for Necrotizing Fasciitis (LRINEC) score has been suggested, first by Wong et al., as a tool for distinguishing necrotizing fasciitis from other less severe and devastating soft tissue infections. An (LRINEC) score of 6 and above should be carefully evaluated and considered for the diagnosis of NF (domains made of total white cell count, hemoglobin, sodium, glucose, serum creatinine, and c-reactive protein) [8].

## Case presentation

A 48-year-old male, known case of hypertension, morbid obesity (BMI: 60), and ischemic heart disease (IHD) post-cardiac stenting one year ago and confirmed to receive coronavirus disease 2019 (COVID-19) vaccine, three doses, presented to the emergency department complaining of dyspnea and right proximal medial thigh pain and swelling with indurated lesion and necrotic patches that started two days ago prior to his presentation, averaging 20 × 20 cm associated with crepitus and warmth and reaching up to the perineum.

**Figure 1:**
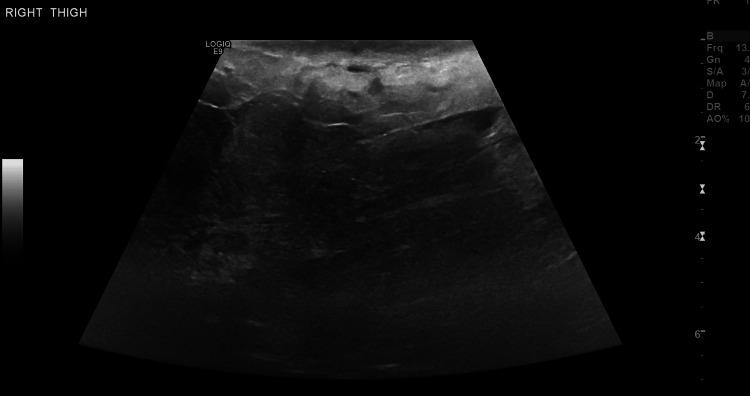
Patient's US at presentation to our hospital. The study was limited due to body habitus, however, there is diffuse subcutaneous edema.

The dyspnea and swelling got worse over the previous night, for which he sought medical attention in another hospital. He was found to be afebrile but tachypneic, tachycardic, and toxic looking. For this, we did angiographic computed chest tomography to rule out pulmonary embolism which came out to be negative. Next, we did a thigh ultrasound which showed diffuse subcutaneous edema of the proximal right inner thigh (Figure 1). The patient decided to leave after that and presented to the emergency department at our institution the next day.

**Figure 2:**
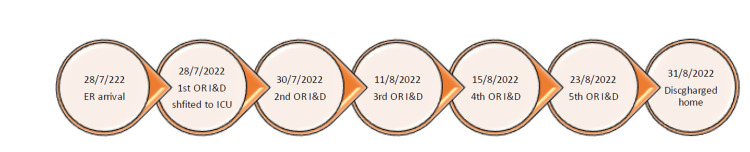
Patient's admission timeline. I&D: irrigation and debridement

Upon presentation to our hospital, patient was found to be still afebrile but toxic appearing and hemodynamically unstable with tachycardia, tachypnea, and severe hypotension, not responding to adequate fluid resuscitation (Figure 2). Patient was then given phenylephrine with no response as well and was next started on maximum peripheral norepinephrine, but he continued to be hemodynamically unstable too. Central line was inserted, and the intensive care team was brought on board and they were able to maintain his blood pressure after adding another vasopressor. The Glasgow Coma Scale upon presentation was 15/15 and the patient was sating well on room air despite being tachypneic. Immediately after that, the patient was started on broad-spectrum triple intravenous antibiotics (meropenem, vancomycin, and clindamycin).

Relevant laboratories and inflammatory markers upon presentation were as follows: WBC 24.5 µL, c-reactive protein (CRP) 412 mg/L (normal less than 5), erythrocyte sedimentation rate (ESR) 63 mm/h (normal less than 20), procalcitonin 89.7 µg/L (normal 0.29 - 3.40), pH arterial 7.25 (normal 7.35 - 7.45), pH venous 7.30 (normal 7.32 - 7.43), keeping in mind that arterial pH dropped to 7.14 with base excess ± 12 and HbA1c was 6.5 (Table 1).

**Table 1:** Labs during and after patient receiving treatment.

Labs.	Upon admission	4 weeks post-treatment	Upon discharge
White blood cells (WBC)	24.5 µL	16 µL	7.2 µL
Erythrocyte sedimentation rate (ESR)	63 mm/h	52 mm/h	29 mm/h
C-reactive protein (CRP)	412 mg/L	92 mg/L	11 mg/L
Lactic acid	4.6 mmol/L	Normal	Normal
Hemoglobin	11.5 g/dL	-	-
Sodium	137 mmol/L	-	-
Creatinine	290 umol/L	-	-
Glucose	6.6 mmol/L	-	-

**Figure 3:**
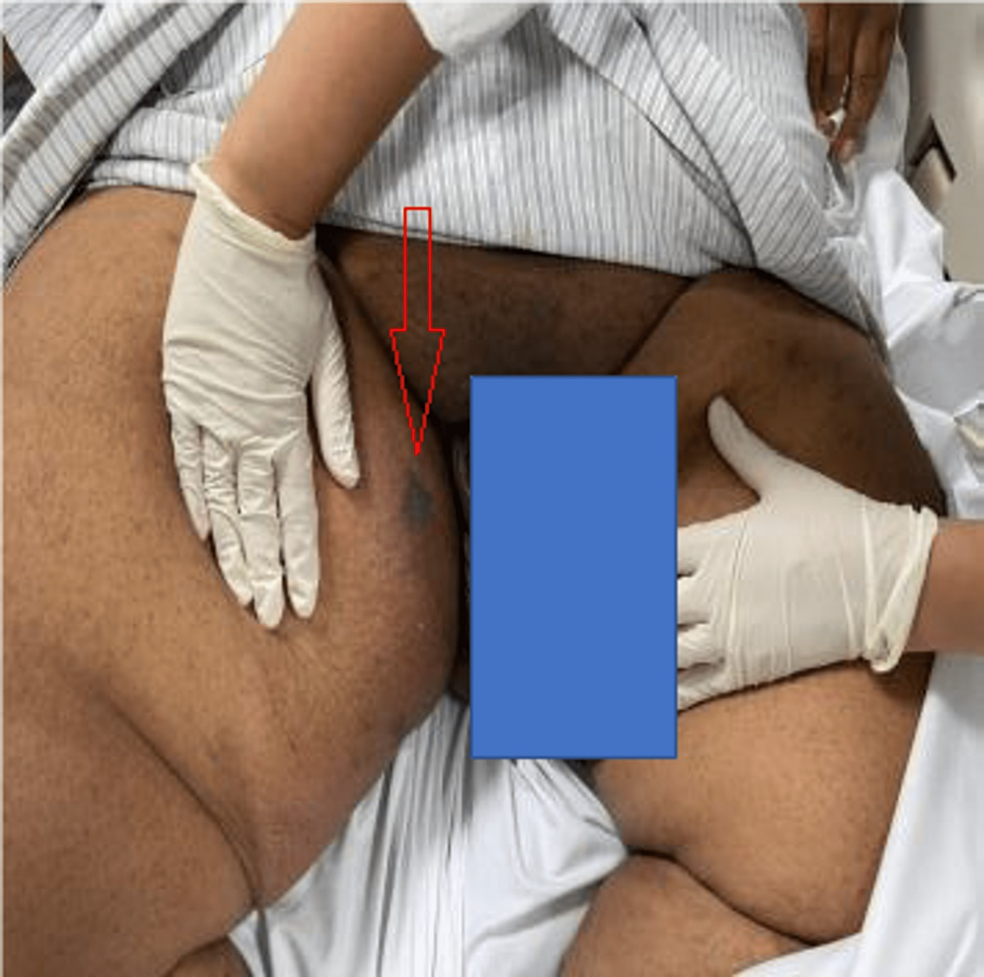
Initial clinical presentation at the emergency department.

**Figure 4:**
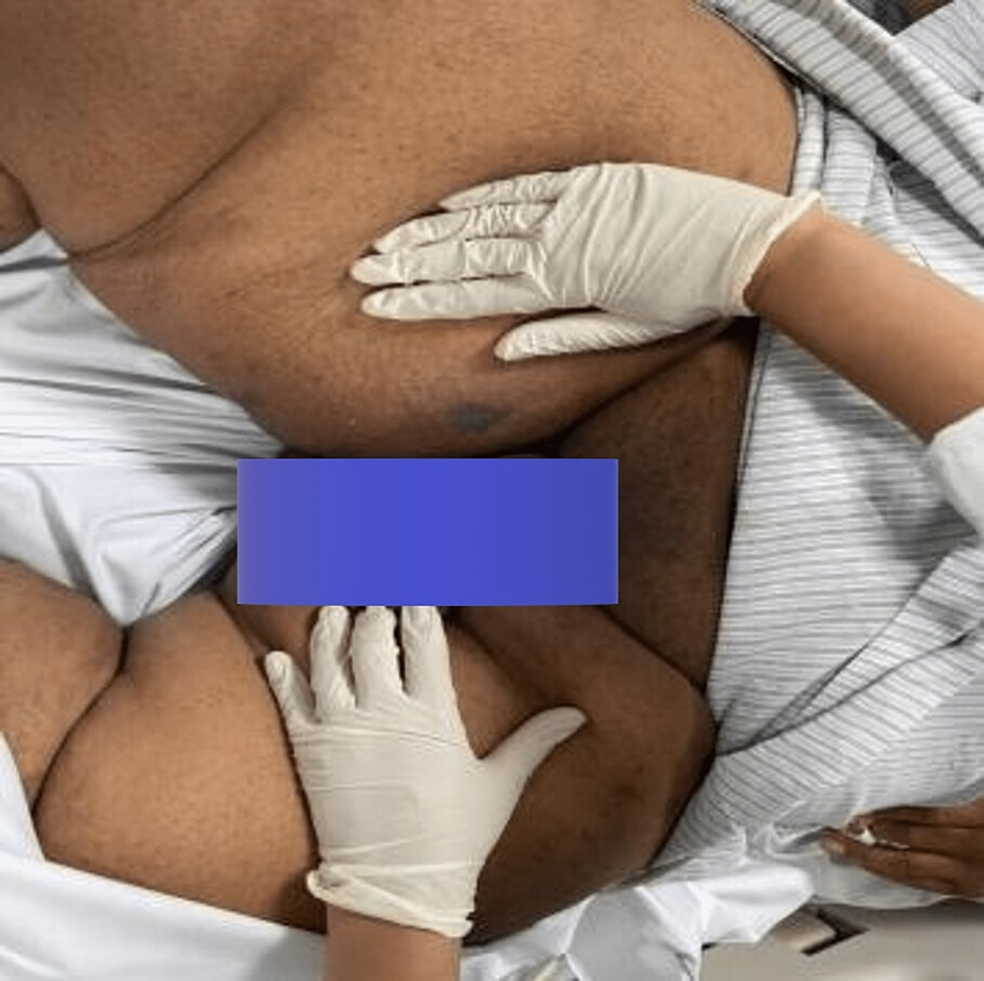
Another initial clinical presentation at the emergency department.

A swelling extending from the right medial mid-thigh all the way to the groin is noted, measuring 20 x 20 cm, warm with erythema and induration over it, no bullae could be observed, but there was an extremely tender area of distinct blackish/silver discoloration over the proximal aspect of the inner thigh and there was associated crepitus all over the swelling (Figures 3, 4).

**Figure 5:**
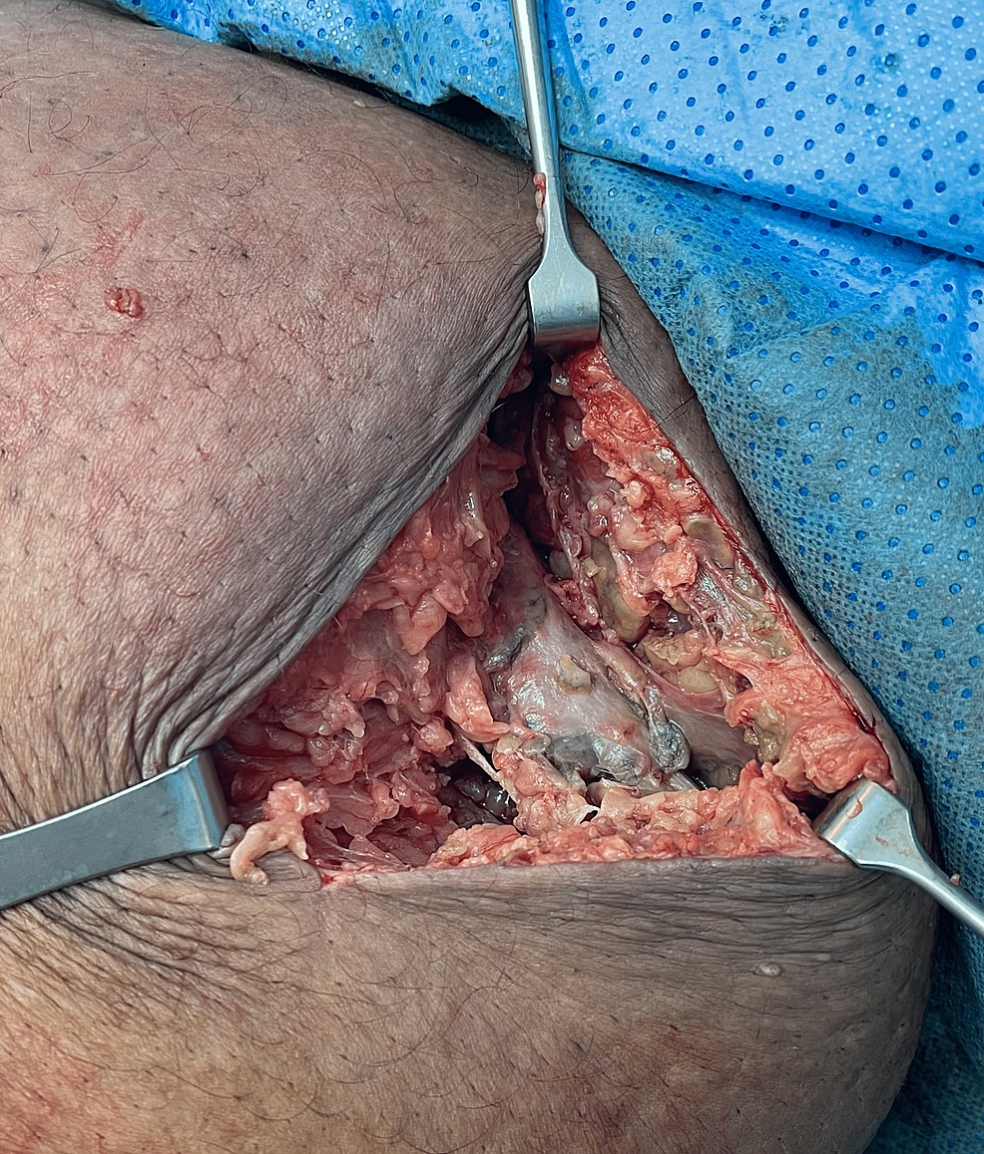
First surgical irrigation and debridement (intraoperative pic) #1.

**Figure 6:**
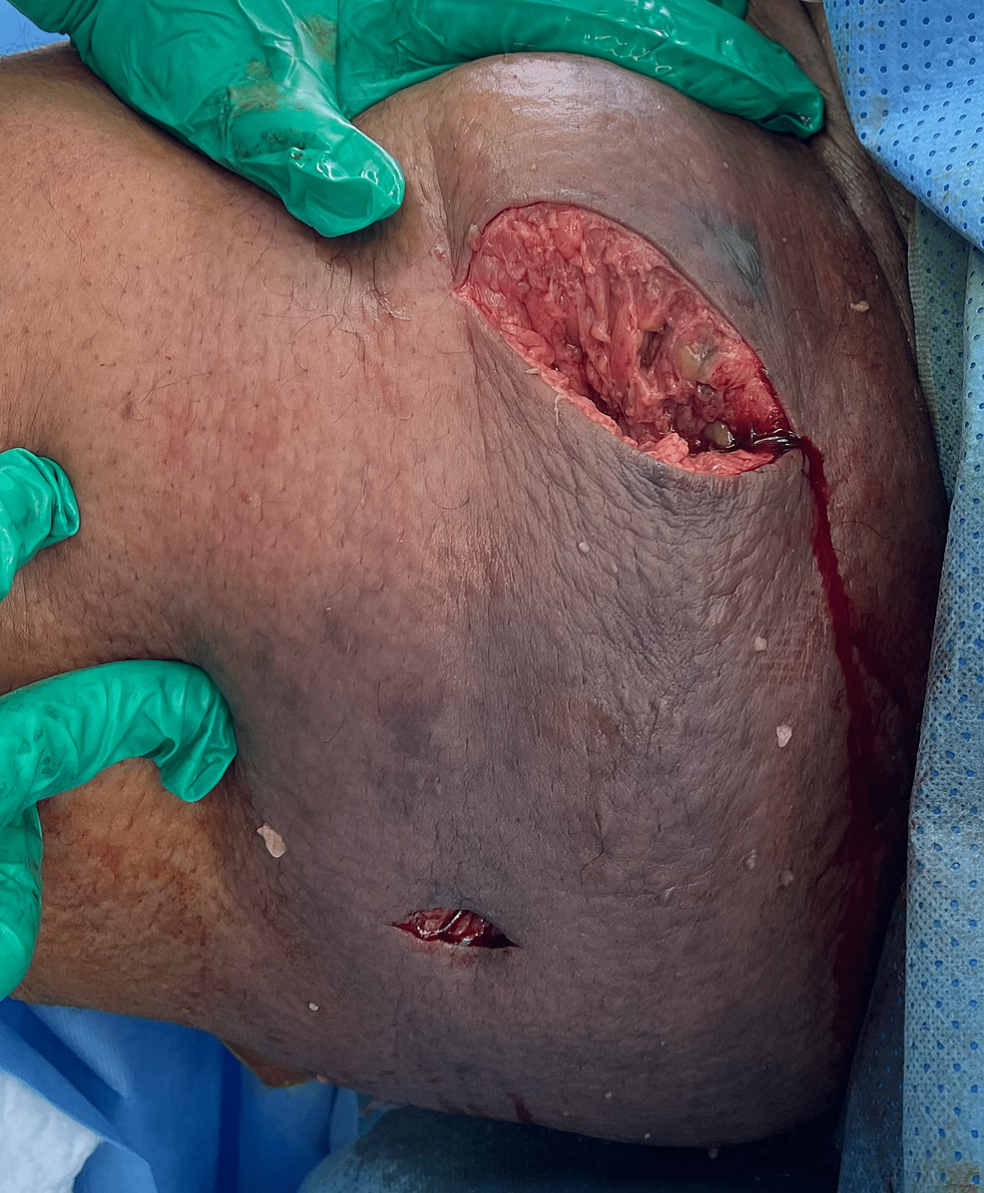
First surgical irrigation and debridement (intraoperative pic) #2.

**Figure 7:**
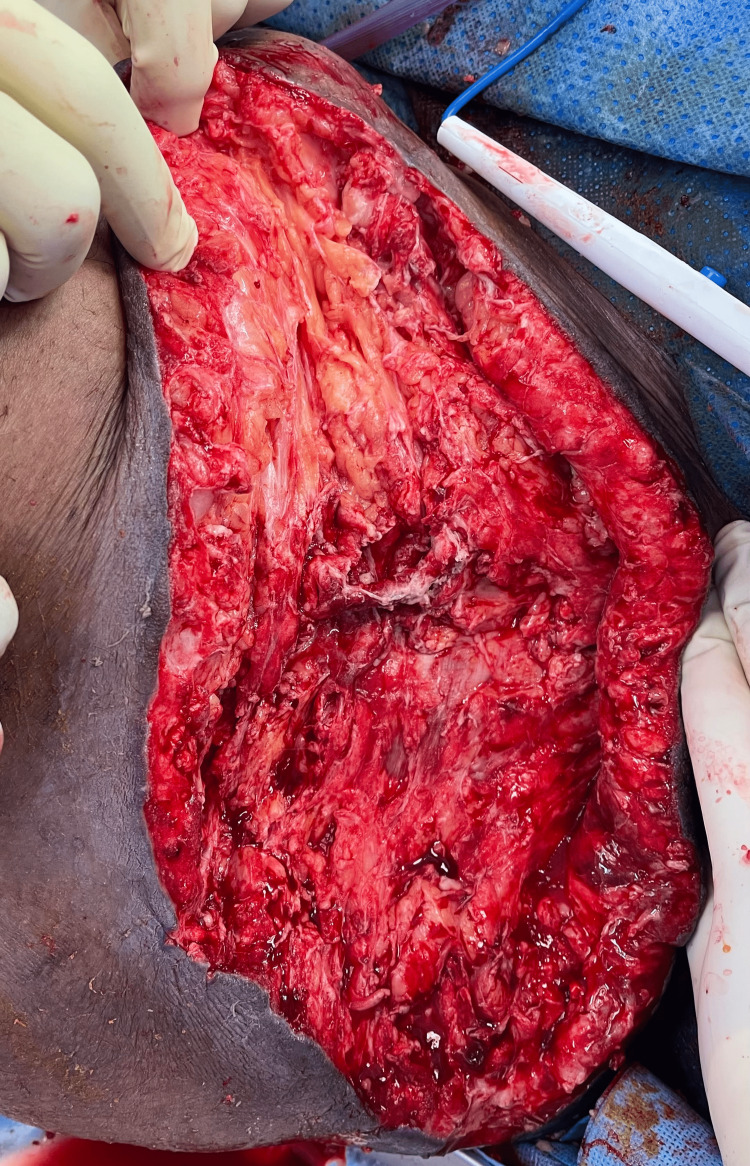
First surgical irrigation and debridement (intraoperative pic) #3. Extensive radical debridement was done, removing all the devitalized necrotic tissues, debridement reached down to the posterior thigh and proximally just short of the groin.

Immediately after that, the patient was transferred to the operating room for emergent radical extensive irrigation and debridement (Figures 5, 6, 7). Intraoperatively, the skin and tissue under the swelling were found to be non-viable and not bleeding. A distinctive foul-smelling blackish silver-colored superficial and deep tissue was noted. Apart from a small amount of foul-smelling serous discharge along with odorous liquified fatty tissue, there was no specific collection. Also, hard thrombosed vessels were noted in the wound bed. Aggressive debridement was done until healthy bleeding tissue was reached. After that, the patient was admitted to ICU with initial diagnosis of septic shock and kept intubated, sedated, and mechanically ventilated on three different vasopressors. His COVID-19 polymerase chain reaction (PCR) result came back positive along with positive respiratory culture of *Stenotrophomonas maltophilia*. All intraoperative fluid and tissue cultures that were taken from the thigh wound in the first and subsequent irrigation and debridement procedures came back negative with no growth of bacterial or fungal organisms, although few Gram-positive cocci.

**Figure 8:**
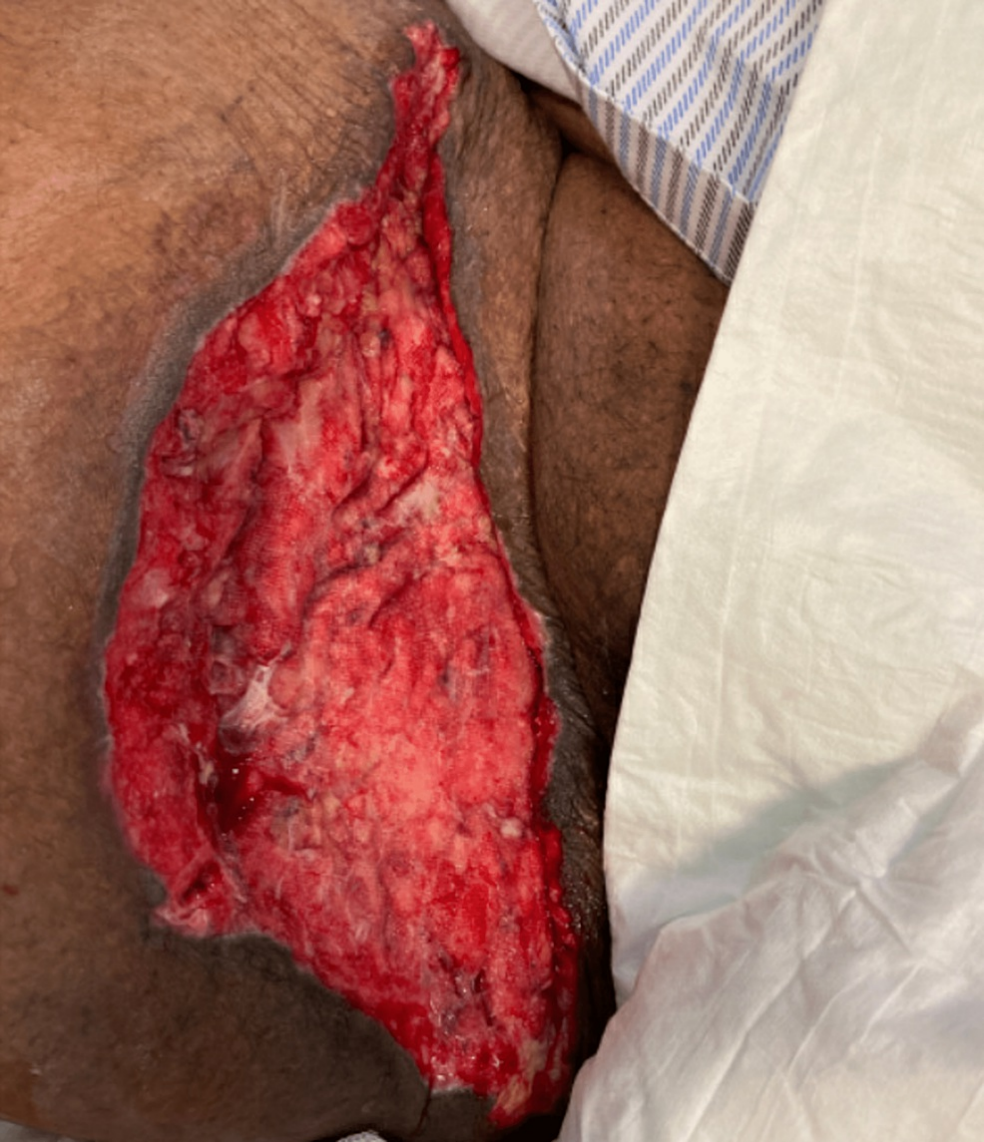
After multiple surgical irrigation and debridement (intraoperative pic). The image shows healthy wound bed with no signs of infection.

**Figure 9:**
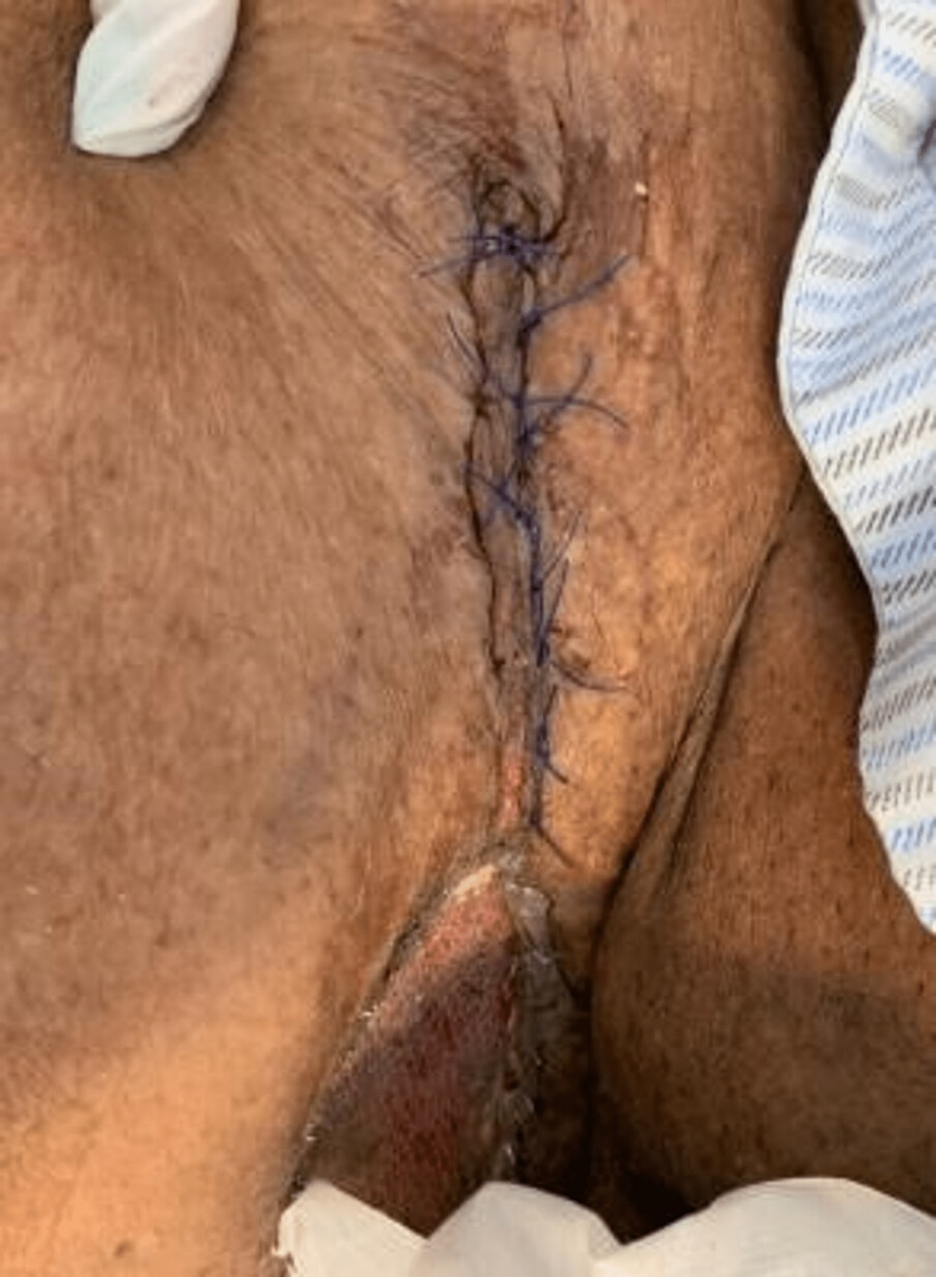
Wound closure in addition to split-thickness graft wound coverage (#2).

**Figure 10:**
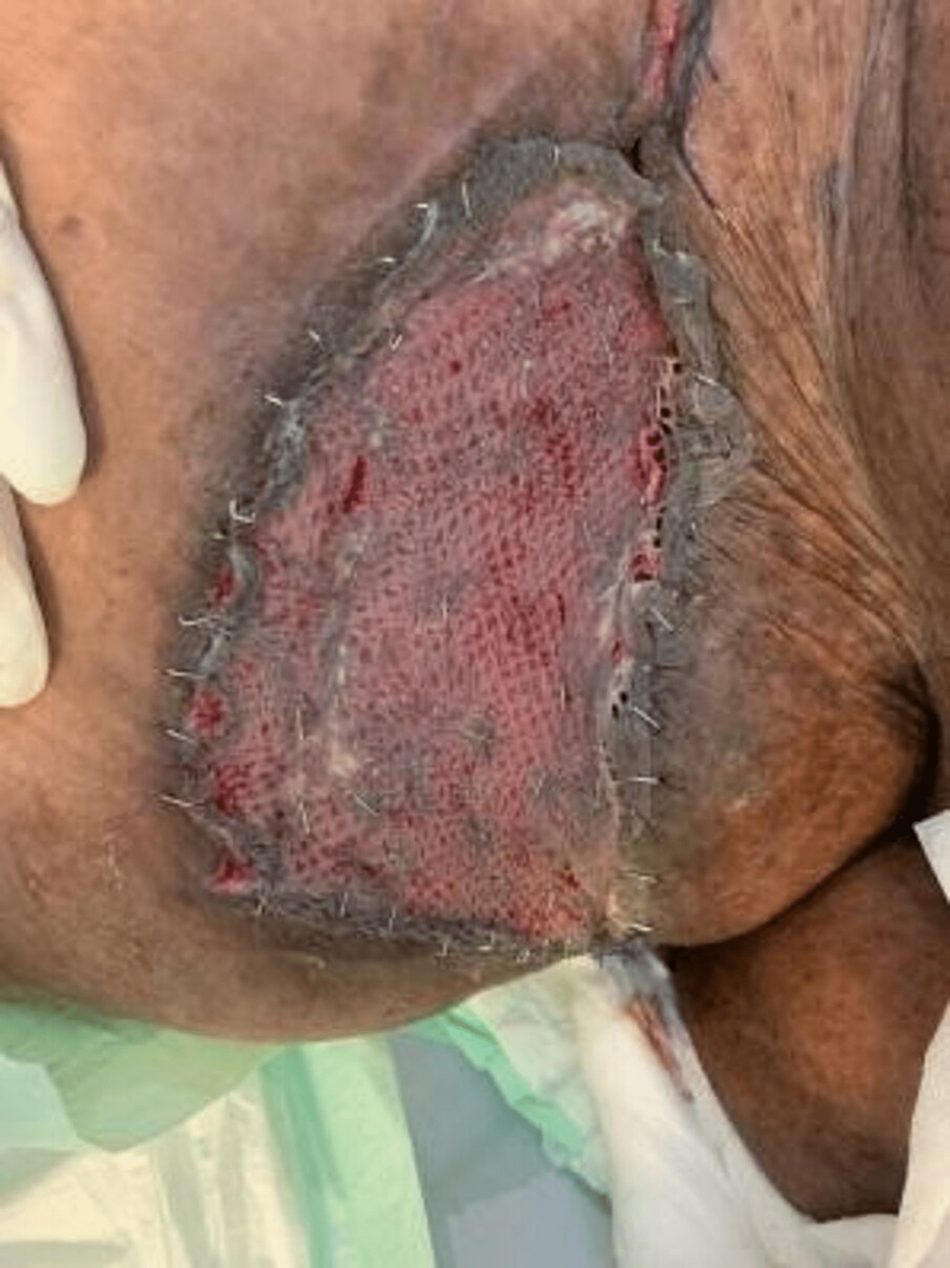
Wound closure in addition to split-thickness graft wound coverage (#1).

And rare Gram-negative bacilli were shown in the initial Gram staining results. Patient's condition initially deteriorated further in ICU and he started to be febrile and developed worsening acute renal failure with anuria, high liver and cardiac enzymes along with atrial fibrillation, and persistent hemodynamic instability. His condition eventually slowly improved with supportive ICU care and he subsequently underwent five more times of irrigation and debridement with vacuum-assisted closure (VAC) and negative pressure wound therapy (NPWT), and latest irrigation and debridement (I&D) (Figure 8). During that period, a bilateral lower extremity venous Doppler ultrasound was done due to swelling and calf pain which showed bilateral proximal femoral and popliteal veins thrombosis. Four weeks later, the patient's condition was more stable with the resolving acute kidney injury and his inflammatory markers were trending down (Table 1). Subsequently, the patient underwent successful wound closure in addition to split-thickness skin graft wound coverage (Figures 9, 10). After five weeks from his initial presentation, the patient was discharged home in stable condition.

## Discussion

Necrotizing fasciitis (NF) is an infectious disease, characterized by rapid bacteria spreading along the fascial planes. It is a rare entity with an increasing number of patients affected in the last few years. In most patients, NF develops in the upper or lower extremities (about 57.8%) [7]. But, cases of cervical or abdominal NF have also been described [9]. Several countries have reported an increase in the NF in the past 20 years, an alarming trend, especially considering the high mortality rate [8,10].

It is generally accepted that predisposing factors to NF include diabetes, alcoholism, tobacco abuse, immunosuppression, malnutrition, advanced age, peripheral vascular disease, renal failure, underlying malignancy, and obesity [11]. Precipitating events of NF include surgery, minor invasive procedures (e.g., joint aspiration and acupuncture), intravenous drug use, penetrating injuries (e.g., insect and animal bites), soft tissue infection, burns, and childbirth [12]. Most cases of NF occur in the abdomen, groin, and extremities. NF in the head and neck region is rare, with most cases having an odontogenic origin [11].

Diagnosis of NF can be challenging as it is a rare entity, and especially peculiar in our patient was his equivocal presentation, as there were no obvious classical pointers or familiar necrotizing fasciitis aggressive soft tissue involvement along with his hemodynamic instability that precluded any further investigation or imaging modality prior to taking the patient urgently to OR. Also, of great importance is the marked hemodynamic instability as compared to the patient fairly moderate early limb involvement. And although COVID-19 infection complicated the presentation and hospital course of this patient, it is unclear how those two clinical entities intersected to produce this devastating and unusual fulminant clinical picture.

Recently, criticism has been made regarding (LRINEC) scoring system prognostic value, especially in patients who already have confounding comorbidities at presentation that can affect domains like creatinine and inflammatory markers [13,14]. In the case we presented, although the (LRINEC) score was 8, we believe that COVID-19 co-infection upon presentation played a confounding diagnostic factor. Also in this case, the patient after first surgical irrigation and debridement was still unstable and in septic shock and had to be shifted to ICU where he stayed intubated, sedated, and mechanically ventilated due to septic shock complicated by severe respiratory symptoms of COVID-19 infection including pneumonia. We believe that the severity of the sepsis and the patient persistent hemodynamic instability was in part resulting from the COVID-19 co-infection.

Adding to the difficulty in diagnosing necrotizing fasciitis in this case and raising questions regarding the cause of the severe sepsis and persistent hemodynamic instability is the fact that all tissue and blood cultures came back negative. It can be expected, as reported in the literature, that the causative organisms in such presentation, an immunocompromised patient with multiple comorbidities, are usually polymicrobial. But we should keep in mind that idiopathic NF with no organism identified can be seen and that’s why high clinical suspicion must always be present [5].

Also, of interest is the fact that at the early onset of infection, it is difficult to differentiate NF from cellulitis or other common superficial infections. We believe that the overlap between cellulitis and necrotizing fasciitis is more so evident in this case, given the lack of the so familiar fulminant NF finding upon presentation and intraoperatively. We also believe that the aggressive radical extensive debridement done early and repetitively for this patient along with the supportive ICU care and aggressive broad-spectrum anti-microbial therapy helped in bringing this patient from the brink of fulminant refractory septic shock and, and its sequelae, end-stage organ failure that he already was heading towards.

Still of great interest is the atypical and early presentation of this case, along with the rapid and severe deterioration that the patient eventually recovered fully from. In addition to the fact that it was a culture-negative necrotizing fasciitis in its early phase complicated by severe sepsis in a COVID-19-infected patient. This led us to report this case in the literature in hope of shedding some light to help in the early diagnosis and treatment of such presentations.

## Conclusions

Necrotizing fasciitis (NF) can be a devastating infection and leads to death if not recognized early. One must keep in mind the myriad factors that play a role and complicate the diagnosis and eventually the treatment of such cases. Having a high clinical suspension of NF when dealing with patients at risk or those with equivocal presentation can lead to reduced morbidity and mortality rates. Early diagnosis of NF can lead to early surgical intervention, and this along with appropriate supportive measures can dramatically improve patient outcomes.

## References

[1] Sarani B, Strong M, Pascual J, Schwab CW (2009). Necrotizing fasciitis: current concepts and review of the literature. J Am Coll Surg.

[2] Kim YH, Ha JH, Kim JT, Kim SW (2018). Managing necrotising fasciitis to reduce mortality and increase limb salvage. J Wound Care.

[3] Koh TH, Tan JH, Hong CC, Wang W, Nather A (2018). Early clinical manifestations of vibrio necrotising fasciitis. Singapore Med J.

[4] Heijkoop B, Parker N, Spernat D (2019). Fournier's gangrene: not as lethal as previously thought? A case series. ANZ J Surg.

[5] Wong CH, Chang HC, Pasupathy S, Khin LW, Tan JL, Low CO (2003). Necrotizing fasciitis: clinical presentation, microbiology, and determinants of mortality. J Bone Joint Surgery Am.

[6] Dapunt U, Klingmann A, Schmidmaier G, Moghaddam A (2013). Necrotising fasciitis. BMJ Case Rep.

[7] Angoules AG, Kontakis G, Drakoulakis E, Vrentzos G, Granick MS, Giannoudis PV (2007). Necrotising fasciitis of upper and lower limb: a systematic review. Injury.

[8] Wong CH, Khin LW, Heng KS, Tan KC, Low CO (2004). The LRINEC (Laboratory Risk Indicator for Necrotizing Fasciitis) score: a tool for distinguishing necrotizing fasciitis from other soft tissue infections. Crit Care Med.

[9] Roujeau JC (2001). Necrotizing fasciitis. Clinical criteria and risk factors. [Article in French]. Ann Dermatol Venereol.

[10] Puvanendran R, Huey JC, Pasupathy S (2009). Necrotizing fasciitis. Can Fam Physician.

[11] Chelsom J, Halstensen A, Haga T, Høiby EA (1994). Necrotising fasciitis due to group a streptococci in western Norway: incidence and clinical features. Lancet.

[12] Peetermans M, de Prost N, Eckmann C, Norrby-Teglund A, Skrede S, De Waele JJ (2020). Necrotizing skin and soft-tissue infections in the intensive care unit. Clin Microbiol Infect.

[13] Neeki MM, Dong F, Au C (2017). Evaluating the laboratory risk indicator to differentiate cellulitis from necrotizing fasciitis in the emergency department. West J Emerg Med.

[14] Burner E, Henderson SO, Burke G, Nakashioya J, Hoffman JR (2016). Inadequate sensitivity of laboratory risk indicator to rule out necrotizing fasciitis in the emergency department. West J Emerg Med.

